# Intersection between health, health literacy and local government: a mixed methods approach to identifying ways to better connect people to place-based primary health care in Western Australia

**DOI:** 10.1186/s12913-022-08872-9

**Published:** 2023-01-21

**Authors:** Lynne Millar, Ranila Bhoyroo, Yesid Pineda Molina, Jessica Watts, Antoinette Geagea, Jennifer Murphy, Christina M Pollard

**Affiliations:** Population Health, Curtin University, GPO Box U1987, Kent Street, WA 6845 Perth, Australia

**Keywords:** Local government, Primary health care, Group model building, Health literacy, Community, Place based

## Abstract

**Background:**

The aim of this year-long mixed methods research was to examine the intersection between health, health literacy and local government to identify ways to better connect people to place-based primary health care (PHC).

**Methods:**

Four local government areas located within the Perth metropolitan geographic area provided the setting for the current research. Researchers were co-located into the four local governments over a 10-month period to engage with community stakeholders and services. Two methodologies were used to achieve the objective: eight group model building (GMB) workshops were conducted with *N* = 148 participants to create causal loop diagrams of the barriers and enablers to people being healthy and well in each of the LGAs and develop potential action ideas from these. Surveys were used to collect health service use and health literacy, as measured using a validated Health Literacy Questionnaire (HLQ), across the four LGAs (*N* = 409, approximately 100 respondents/area).

**Results:**

The causal loop diagram themes common across LGAs included: (1) mental health; (2) access to services; (3) health system capacity; (4) economics; and (5) physical wellbeing. Health literacy was relatively high for all nine domains of the HLQ. In the five domains rated from one to four the lowest score was 2.8 for ‘appraisal of information’ and the highest was 3.2 for ‘feeling understood and supported by healthcare providers’. In the four domains rated from one to five; the lowest score was 3.7 for ‘navigating the healthcare system’ and the highest was 4.1 for ‘understand health information well enough to know what to do’.

Prioritised action ideas recommended increases in practitioners to meet local needs and training General Practitioners and other health staff in culturally sensitive and trauma informed health care. The survey findings and field notes from the GMB were used to construct personas embodied in vignettes highlighting general themes identified in the workshops including those relevant to local areas.

**Conclusions:**

There are many possibilities for health care and local governments to work together to bring services to community members disengaged from the health system. Bringing together people from diverse backgrounds and organisations created synergies that resulted in novel and feasible potential strategies to improve community health.

## Background

Ageing populations, longer life expectancy, and increased prevalence of chronic disease and complex comorbidities place a burden on health care services and contribute to rising health care costs in Australia This includes expenditure on health goods and services such as hospitals, primary health care (PHC), referred medical services, research, and capital expenditure [1].

PHC is the front line of the health care system and often the first point of contact for a person. Effective PHC can help avoid unnecessary hospitalisations and improve health outcomes [2]. However, expenditure has shifted away from PHC to hospitals over the last 20 years, from 37% of total health budget due to PHC and 36% due to hospitals in 2000/1 to 34% on PHC and 40% on hospitals in 2017/18 [1]. It is delivered across a variety of settings that do not require a referral and includes a range of practitioners for example, general practitioners (GP), and other allied health practitioners and health professionals (i.e. pharmacists, dentists and Aboriginal and Torres Strait Islander health workers and practitioners pharmaceutical, and community and public health services) [3, 4].

Health professionals deliver PHC services in various settings, including allied health practices, community health centres, general practices, and through communication technology with the emerging use of ‘telehealth’ and online health information [5] websites such as Healthdirect (Government website for free Australian health advice) [6] playing increasing and integral roles. Since 2020 and the onset of the COVID-19 disease pandemic, the Australian Government expanded Medicare-subsidised telehealth services so that Australians could access essential PHC from their home [7].

Hospital systems are under pressure due in part to the prevalence of preventable non-communicable diseases (NCDs), increasing hospital waiting lists, higher patient co-payments, rising private health insurance premiums and increasing out-of-pocket health-related expenses [8]. Locally delivered, place-based PHC can increase timely access to essential services reducing pressure on hospital wait lists and increasing the efficiency and efficacy of patient care. Place-based primary care works by integrating and coordinating government programs and services and incorporating the resources and ideas of local residents, government, service providers and businesses to improve equity in access and accessibility to health services within a geographical area [9]. However, the onus still often falls to the client or patient to seek out these services.

Health literacy (HL), the wide range of skills needed to manage one’s health, plays a key role in the ability to access health care. HL includes the consultation, engagement and communication with healthcare providers and navigation through complex healthcare systems [10–12]. The concept of HL also includes the critical appraisal of health information from different sources, identifying social supports needed to access services and maintain health, keep abreast of available services, and understanding ones’ consumer rights to healthcare [10–12]. Lower HL levels are associated with less knowledge of individual’s health problems [13, 14], how to effectively self- manage [15], lower uptake of health screenings [16] and engagement in health promoting behaviours [17], poor medication adherence [18], as well as higher rates of hospitalisation [19–21], readmission after discharge [22], and poorer overall health [17]. HL is vital to timely access to primary care but many Australians report low levels (59% in 2006) [23].

Access can be defined broadly as the capacity of people to obtain appropriate services in response to need for care. It represents a fit between patient needs and services that meet those needs, or the dynamic interaction between supply and demand [24]. This has been conceptualised by Levesque and Harris’ (2013) whose framework illustrates the active relationship between health consumers and characteristics of the health service providers: ability to perceive, ability to seek, ability to reach, ability to pay and ability to engage and the services’ approachability, acceptability, availability and accommodation, affordability, and appropriateness [25]. Barriers to access can occur due to problems with the abilities of individuals or the attributes of services [10].

There is a dearth of evidence on integrating place-based primary care within local government settings. Local governments are considered well-positioned to engage with communities and lead local planning and initiatives that can influence equity in health outcomes [25]. Little is known about West Australians’ perceptions and understanding of place-based primary care and health literacy.

This study aims to:

- Examine the intersection between health and local government authorities to identify ways to better connect people to place-based PHC.
- Better understand touch points for health in local government, particularly those related to Local Government Frameworks and regulation, for example the Public Health Act (2016).
- Identify opportunities for localised reciprocal referral pathways between community organisations and PHC services.

## Methods

The research was undertaken in three interlinked phases:

1. Engagement, with a project officers housed within four West Australian LGAs to facilitate an understanding of local services and facilities and to identify community organisations with potential to support in-place PHC.
2. Group Model Building (GMB), a systems science participatory approach which brings relevant stakeholders together to identify connections and factors influencing an issue and to develop and prioritise action ideas to address issues of local importance [26].
3. A survey comprising health service access and use, health literacy assessment of community participants using the Access and Health Literacy Questionnaire (HLQ) [12] to identify levels of HL and factors influencing primary care access, and demographic characteristics.

### Engagement

Project officers were placed at each LGA two days a week for 10 months. They attended local government meetings and events and were introduced to a diverse range of local organisations and services with the potential to support place-based health, for example, culturally and linguistically diverse (CALD), seniors, faith-based and other organisations, mothers’ groups, LGBQI + groups, local markets, and libraries. They recruited participants including local government staff, service providers, community groups, and interested individuals to participate in the research.

### Group Model Building (GMB)

Systems thinking methods have been successfully applied to complex health issues [27–30] to identify the most important cause and effect relationships within a specific system. Causal loop diagrams (CLD) are used to map and model dynamic complexity [26]. Two, three-hour GMB workshops with follow-up meetings were held in each LGA between 27 July and 23 September 2021. The facilitated GMB method engages stakeholders to collectively consider the causes and consequences of a complex problem and build an agreed CLD to illustrates these. Utilising participants’ skills and knowledge, GMB aims to capture perceptions of how a specific issue originated, the underlying causes, and how it can be tackled [29]. The group then works to identify mutually desired outcomes [29]. Data analysis and mapping was undertaken using purpose-built software for Systems Thinking in Community Knowledge Exchange (STICKE) designed by Deakin University. CLDs visually map variables identified by participants and their connections to highlight causality and feedback loops to increase understanding of the problem in context, and to analyse the role of loops within a system. The interconnecting solid lines indicate that the variables move in the same direction and the dashed lines that they move in the opposite direction, the direction of the arrow indicates causality as expressed by participants. The interconnections take the form of balancing loops (indicated by odd number of dashed lines in the loop; for example, if a loop contains four variables joined by three solid lines and 1 dashed line, this means that, the three variables with the solid lines connecting them move the same direction and one with the dotted line moves in the opposite direction thereby balancing the system. There are no balancing loops in the CLD below) in which system structure resists change, tending toward stasis and reinforcing loops (indicated by zero or even number of dashed lines. See results section for concrete examples), in which changes in a variable are intensified, resulting in exponential growth or decay[31]. GMB workshop methodology was designed using Scriptapedia “Scripts for Using in Group Model Building Workshops”[32].

The first workshop asked participants collectively to reflected on the question “What are the barriers and enablers to people being healthy and well in the [local government area]?” Across the two GMB workshops, participants:

- Identified the PHC services and facilities in their LGA.
- Identified the barriers and enablers to people being healthy and well in the community.
- Created a CLD of the enablers and barriers to PHC.
- Formulated and prioritise action ideas that could be undertaken.

Presentations, small group discussions and a series of activities were used to develop the CLD. Field notes and written information were used by the modelling team to refine the CLD between workshops one and two. The draft model was then presented to participants for review in the second workshop after which is was finalised and action ideas were then brainstormed and prioritised. Follow-up meetings that aimed to gauge the appetite for developing and implementing action ideas were held.

A final CLD was constructed by LM and CMP at the end of the project to illustrate the variables and themes common to all local government areas to aid translation of findings and generalisation to other local government areas in Western Australia. This method also served to preserve anonymity of individual LGAs.

### Access and health literacy survey

Health literacy is vital for people to be able to access appropriate and timely health care. The validated Health Literacy Questionnaire (HLQ) was used to measure health literacy [12]. Nine scales and 44 items capture a wide range of the lived experiences of people attempting to engage in understanding, accessing and using health information and health services as well as reflections on the quality of health and social service provision [12].The HLQ assess perspectives on being understood and supported by healthcare providers, having sufficient information to manage my health, actively managing their health, social support for health, appraisal of health information, ability to actively engage with healthcare providers, navigating the healthcare system, ability to find good health information and understand health information well enough to know what to do. Responses for each domain are averaged to produce nine continuous output data items for analyses. The final instrument included 62 items measuring socio-demographics and assessment of issues relating to access and availability of health services as identified through GMB workshops (e.g., transport limitations, difficulty accessing services, financial barriers to PHC).

Purposeful snowball sampling was used to recruit community members, local government staff or service providers aged 18 years and over who resided within each LGA. Methods included emails, posters, and personal approaches at local community facilities, groups, and events. The survey was paper-based or online (Qualtrics) sent via an email link or a QR code on personal electronic devices or IPads provided by the research team conducted between 30 September to 15 November 2021. One AUD$100 Coles/Myer voucher was offered in each LGA in a prize draw for those who had completed the survey.

Informed consent to participate was obtained from all participants. Ethics approval for this research was sought and obtained from the Curtin University Human Research Ethics Committee, approval number HRE2021-0414. All methods were performed in accordance with the relevant guidelines and regulations (for example- Declarations of Helsinki) [33].

Descriptive statistics and hierarchical cluster analysis based on the nine health literacy domains used Ward’s linkage and the squared Euclidean distance as recommended by the Ophelia manual methodology [16, 34, 35] were used to analyse the survey data. A total of three to16 clustering solutions were specified for the analysis and missing data were excluded. Profiles of the cluster analysis were then combined with demographics to provide a detailed picture of the characteristics of each profile with each participant assigned to only one profile.

## Results

### Common variables and themes across all LGAs

**Fig. 1:**
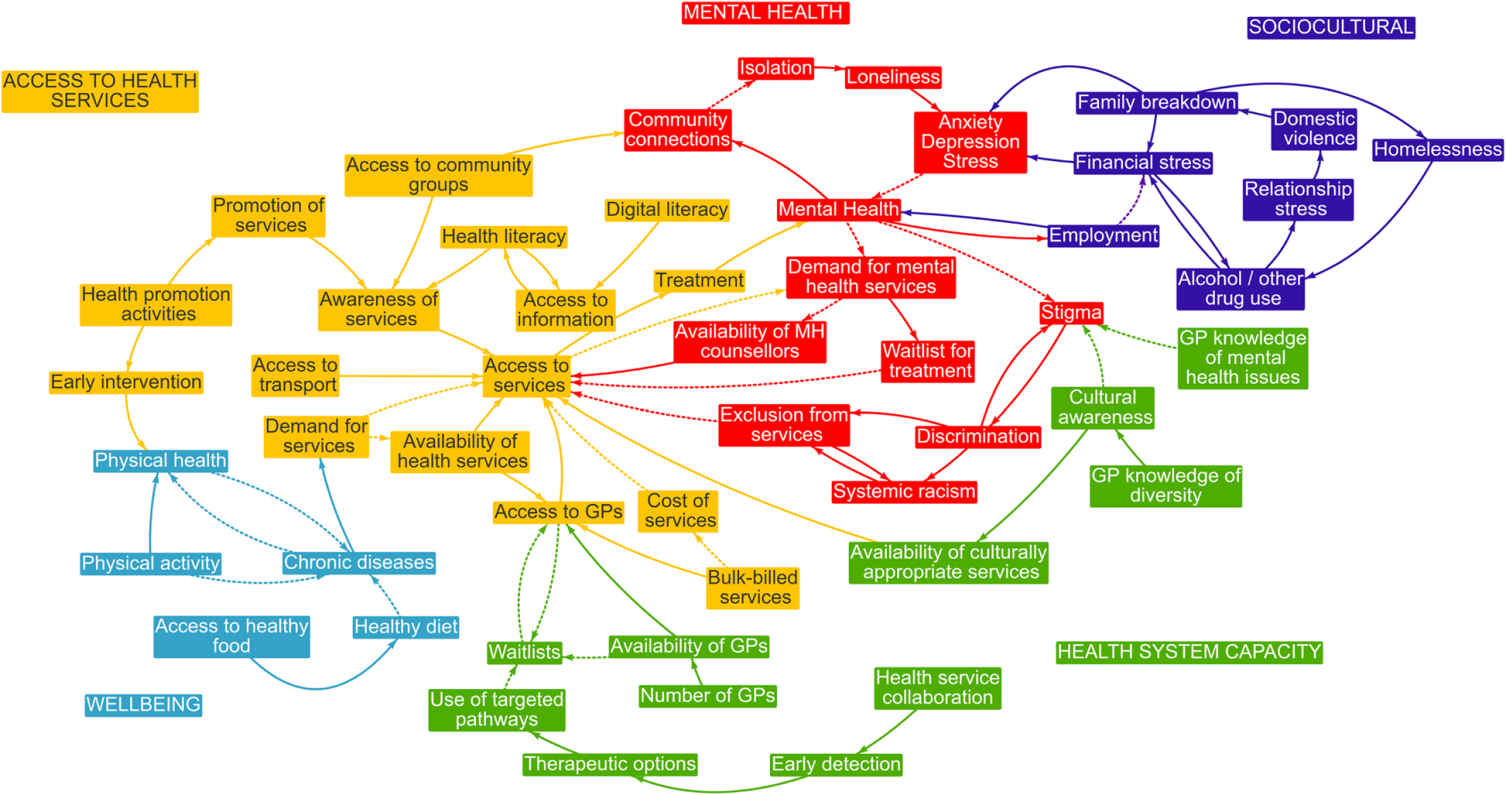
Causal Loop Diagram of the common themes, barriers, and enablers to people being healthy and well in four local government areas

The CLD themes common across LGAs included: (1) mental health; (2) access to health services; (3) health system capacity; (4) sociocultural; and (5) physical wellbeing. See Table 1 for a summary of the themes and factors associated with them. Figure 1 shows a CLD of the common variables and themes across all four local government areas.

**Table 1:** Common themes, barriers, and enablers to people being healthy and well in LGAs identified in CLD

**Variables in common **	**LGA1**	**LGA2**	**LGA3**	**LGA4**
**Mental health**				
Stigma	x	x		x
Discrimination/Racism/Ageism	x	x	x	
Suicide	x	x		
Isolation	x	x	x	
Community connections	x	x	x	x
Loneliness	x		x	
GP awareness of mental health issues	x		x	
Anxiety/depression/stress			x	x
**Access**				
Access to primary care services	x	x	x	x
Awareness of services	x	x	x	x
Access/availability of transport	x	x	x	x
Disability/NDIS services access	x	x	x	
Access to information	x	x	x	
Translators/interpreters	x	x		
Availability/access of culturally appropriate health services	x	x		
Age-appropriate health services	x	x		
Distance to services	x		x	
Digital literacy		x	x	
**Health system capacity**				
Waitlists	x	x	x	x
Early detection/intervention	x		x	x
Awareness of needs of Aboriginal & Torres Strait Islander people	x	x		
Health services collaboration/coordination	x		x	
Number of health practitioners			x	x
Availability of health services	x		x	
**Sociocultural**				
Financial stress	x	x	x	x
Domestic violence	x	x	X	
Alcohol and other drugs	x		x	x
Employment		x	x	x
Medicare gap and other service payments	x	x		x
Homelessness	x	x		x
Family breakdown/relationship stress	x		x	
**Wellbeing**				
Access to healthy food	x	x		x
Fast food availability and consumption		x	x	x
Physical activity	x	x	x	
Backyard space for play	x	x		
Exercise space	x	x		
Chronic diseases			x	x
Holistic approach to wellbeing		x	x	
Time pressure	x		x	

Stigma surrounding mental health problems was described as a key barrier to service usage and help seeking. Discrimination and racism, particularly towards Aboriginal and Torres Strait Islander people, and ageism were identified barriers to accessing health services. A lack of mental health knowledge and cultural awareness among GPs impacted access to services through a pathway from stigma to discrimination to systemic racism to exclusion from services, see Fig. 1 illustrating the mental health theme. Participants described a reinforcing loop that started with levels of mental health; as mental health decreased this increased the demand for mental health services, and, as demand increased, so did the waitlists which led to a decrease in access to services, which in turn led to a decrease in treatment, and a subsequent decrease in mental health. Another reinforcing loop illustrated the impact of community connections on mental health via isolation and loneliness, and anxiety, depression, and stress.

Access to, and awareness of, health services and other organisations generally, and more specifically culturally and age-appropriate services, were described as impacting the health of community members. Health literacy was mentioned explicitly by one group and implied through discussions about the lack of awareness and knowledge of, and access to, health services. Inability to afford allied health services or pay the Medicare gap were described. There was a perception that the difficulty accessing GPs was because there were not enough practitioners in the local area. The lack of, or appropriateness transport, especially for those people with disabilities and who lived further away from services also limited access.

Health system capacity was a key concern. Waitlists for services described by participants as being due to both the unavailability of services and to the low number of health care practitioners. Participants described the impact on waitlists and levels of health service collaboration which could influence early detection, leading to more therapeutic options and targeted referral pathways. Available GPs were described as lacking awareness of the specific needs of diverse groups; culturally and linguistically diverse, gender, and Aboriginal and Torres Strait Islander, and people living with disability.

Interrelated factors of financial stress, domestic violence, levels of employment, homelessness, family breakdowns, and harmful use of alcohol and other drugs were clustered in a sociocultural theme. Factors influencing wellbeing included diet, physical activity which were seen to influence the prevalence of chronic disease in the community.

### Action ideas

Table 2 shows the action ideas common across all LGAs that were prioritised as potential interventions based on local CLDs.

**Table 2:** Action ideas to promote health and wellness in four local government areas developed using the Causal Loop Diagrams

Theme	LGA1	LGA2	LGA3	LGA4
Increasing mental health treatment	Separate Emergency Departments for mental health presentations		Mental health hub	Increasing mental health services
Bringing holistic health care to the community	Hub-style Outreach Pop-up services	One stop shop for GP, dentist, mental health, Salvation Army Holistic culturally appropriate approach to health care	Wellness centre Collaboration between providers	Collaboration between services Pop-up clinics
Early intervention			Early childhood intervention	Early intervention on mental health, especially dementia
Financial interventions	Improve funding models			Health professionals donating time
Improve knowledge of available health services & work with community	Increased Education/Awareness of available services in local area	Increase access to information Increase awareness of bilingual GPs in LGA	Directory of services	Increase awareness of available services among GPs Promote existing health services
Health promotion activities	Health services expo		Health services expo	
Improve digital literacy		Increasing digital literacy	Intergenerational digital literacy	

### Health literacy

Table 3 shows the sociodemographic characteristics of the *N* = 410 survey respondents, with 75% female, 82% speaking English at home, 34% with high school the highest educational attainment, 43% living with a spouse/partner, 69% able to save some money each week, 41% were aged 65 years or older, and 40% retirees. 98% had Medicare cards, 65% had private health insurance, and 70% visited their GP in the previous three months, 77% waited less than a week after they booked for their appointment. 42% said it was difficult/or very difficult to access after hour medical services. 69% of respondents said they were likely to attend a health check, screening, or service if it was available locally (e.g., at the local library, senior citizens’ centre, sporting club or other community centre).

**Table 3:** Sociodemographic and health characteristics of community survey participants, by LGA

	LGA1 (*n* = 100)		LGA2 (*n* = 119)		LGA3 (*n* = 124)		LGA4 (*n* = 67)		Total (*N* = 410)	
	n	%	n	%	n	%	n	%	N	%
**Gender** (*N* = 410)										
Female	69	69.0	90	75.6	99	79.8	50	74.6	308	75.1
Male	27	27.0	28	23.5	25	20.2	17	25.4	97	23.7
**Age** (years, *N* = 391)										
18–44	36	36.0	25	21.0	39	35.8	24	38.1	124	31.7
45–64	27	27.0	34	28.6	24	22.0	20	31.7	105	26.9
65+	37	37.0	60	50.4	46	42.2	19	30.2	162	41.4
**Current living situation** (*N* = 409)										
Couple	44	44.0	57	47.9	40	32.5	34	50.7	175	42.8
Live alone	23	23.0	29	24.4	44	35.8	6	9.0	102	24.9
Family with children	26	26.0	29	24.4	36	29.3	25	37.3	116	28.4
**Country of birth** (*N* = 406)										
Australia	67	67.7	75	63.0	63	51.2	32	49.2	237	58.4
Overseas	32	32.3	44	37.0	60	48.8	33	50.8	169	41.6
**Language other than English spoken at home** (*N* = 407)										
Yes	9	9.1	12	10.2	44	35.5	8	12.1	73	17.9
No	90	90.9	106	89.8	80	64.5	58	87.9	334	82.1
**Highest educational attainment** (*N* = 404)										
High School	31	31.4	36	30.5	48	39.3	24	36.9	139	34.4
TAFE/Trade	33	33.3	41	34.7	27	22.1	15	23.1	116	28.7
University	35	35.4	41	34.7	47	38.5	26	40.0	149	36.9
**Current employment status** (*N* = 407)										
Paid employment	51	51.5	53	44.9	36	29.0	33	50.0	173	42.5
Unpaid employment	11	11.1	9	7.6	34	27.4	15	22.7	69	17.0
Retired	37	37.4	56	47.5	54	43.5	18	27.3	165	40.5
**Household money situation** (*N* = 397)										
Spend more	24	24.5	28	24.6	43	35.5	28	43.8	123	31.0
Save more	74	75.5	86	75.4	78	64.5	36	56.3	274	69.0
**Self-rated health** (*N* = 405)										
Excellent/very good	47	47.5	41	35.0	54	43.9	27	40.9	169	41.7
Good	36	36.4	57	48.7	39	31.7	22	33.3	154	38.0
Fair/Poor	16	16.2	19	16.2	30	24.4	17	25.8	82	20.2
**Diagnosed medical conditions** (*N* = 402)										
None	42	42.4	29	24.8	48	40.0	27	40.9	146	36.3
One	24	24.2	30	25.6	22	18.3	18	27.3	94	23.4
Two or more	33	33.3	58	49.6	50	41.7	21	31.8	162	40.3

**Fig. 2:**
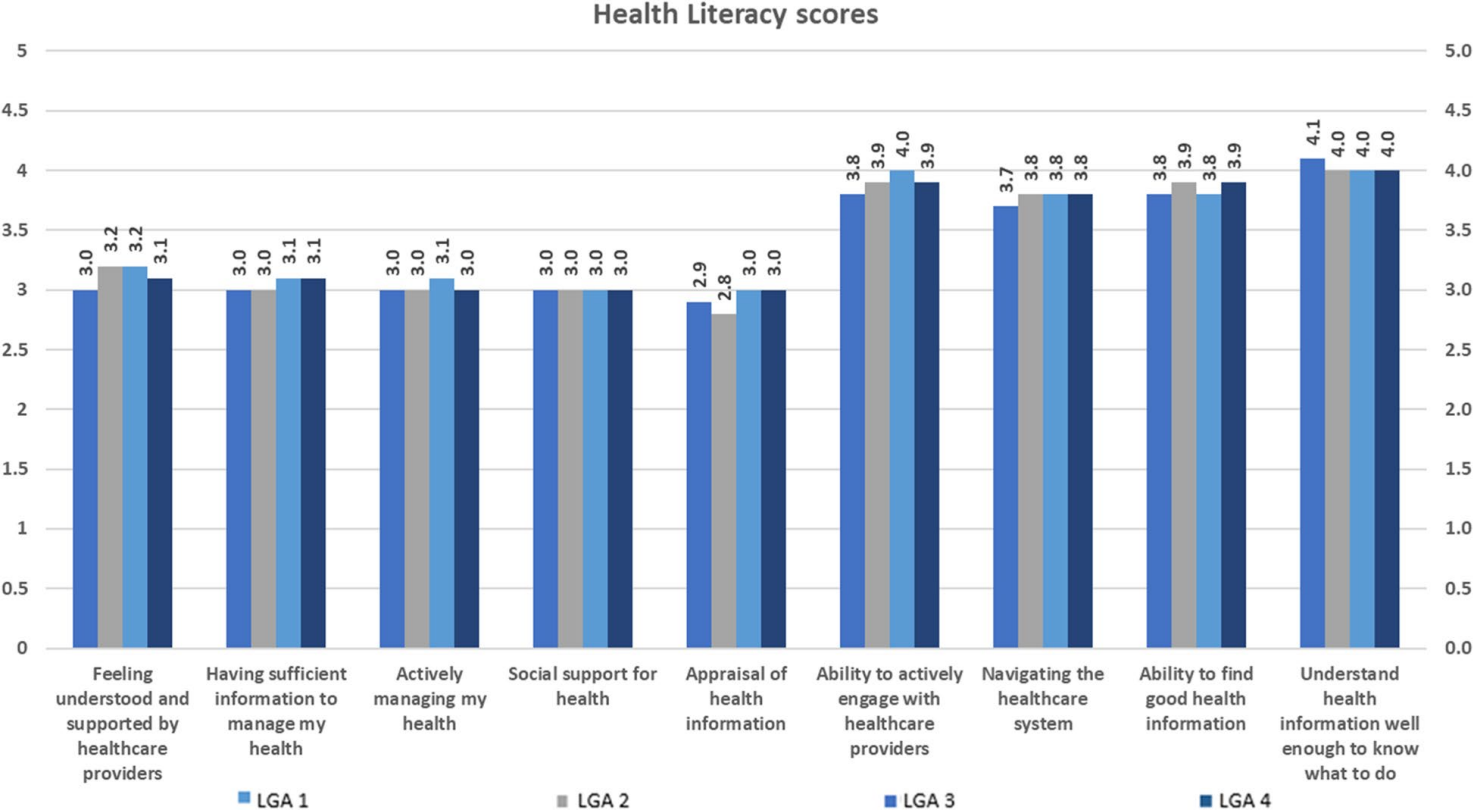
Mean Scores by Health Literacy Questionnaire subscales, by LGAs (*N* = 409)

Figure 2 presents the mean scores of the HLQ with rating of tasks associated with accessing and engaging with service organisations. Five domains were scored out of four and their mean scores ranged between 2.8 and 3.2 (Feeling understood and supported by healthcare providers = 3.0-3.2; Having sufficient information to manage my health = 3.0-3.1; Actively managing my health = 3.0-3.1; Social support for health = 3.0; Appraisal of health information = 2.8-3.0). The other four domains were rated out of five and the mean scores varied between 3.7 and 4.1 (Ability to actively engage with healthcare providers = 3.8-4.0; Navigating the healthcare system = 3.7–3.8; Ability to find good health information = 3.8–3.9; Understand health information well enough to know what to do = 4.0-4.1). Cluster analysis highlighted different patterns and areas of concern, see.

**Table 4:**
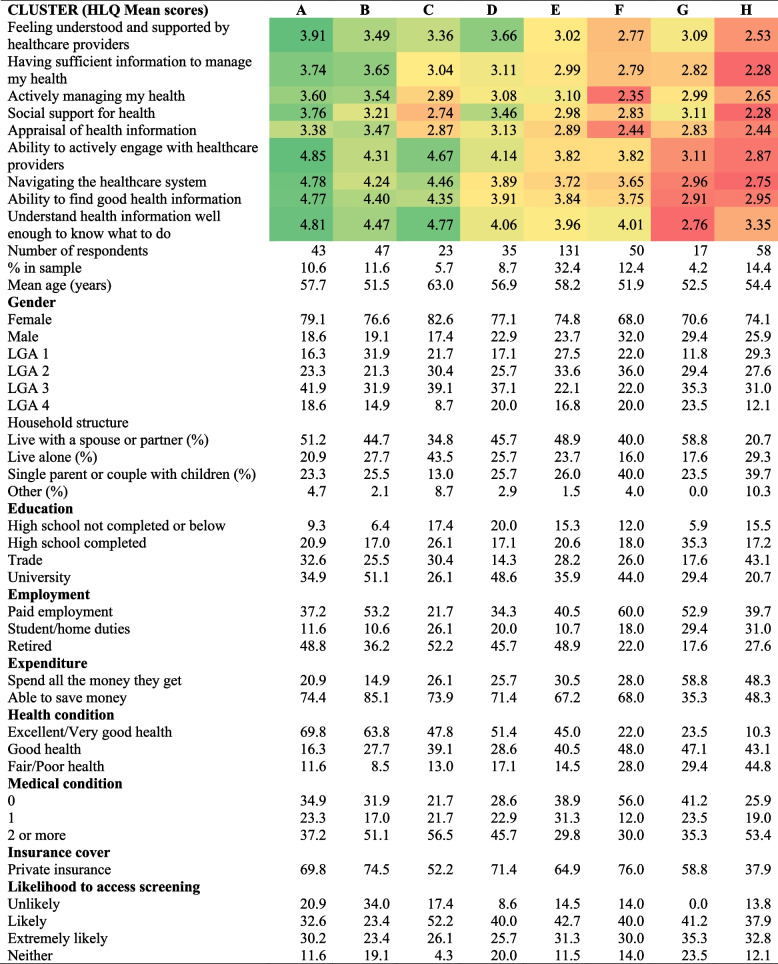
Health literacy profiles of respondents based on an 8-cluster solution

Table 4 presents the eight clusters using a heatmap (from green (highest scores) to red (lowest scores)) of the mean HLQ scale scores and sociodemographic characteristics of each cluster. Clusters A and B had generally higher scores across the nine health literacy scales and six clusters (C to H) had lower scores with differing patterns. People felt understood and supported by healthcare providers, except in clusters F and H, they had sufficient information to manage their health, except in clusters F to H, were actively managing their health, except for clusters C, F to H; had social support for health, except for clusters C, E, F and H, and were able to appraise health information, except for C, E, F to H. People felt able to actively engage with healthcare providers, except for in clusters G and H; were able to navigate the healthcare system, except for clusters G and H; were able to find good health information, except for clusters G and H; were able to understand health information well enough to know what to do, except for clusters G and H. The ability to appraise health information was lower across all clusters.

Demographic characteristics relating to health literacy scores varied: single parents and couple households with children were more likely to be in clusters F to H; students or those who reported undertaking home duties were more likely to be in clusters G and H; retired people were less likely to be in clusters F to H; those unable to save money were more likely to be in clusters G and H; people who reported being of fair to poor health were more likely to be in clusters F to H; and those without private health insurance were more likely to be in cluster H.

The survey findings and field notes from the GMB were used to construct personas embodied in vignettes highlighting general themes identified in the workshops as well as local issues [36]. See Table 5.

**Table 5:**
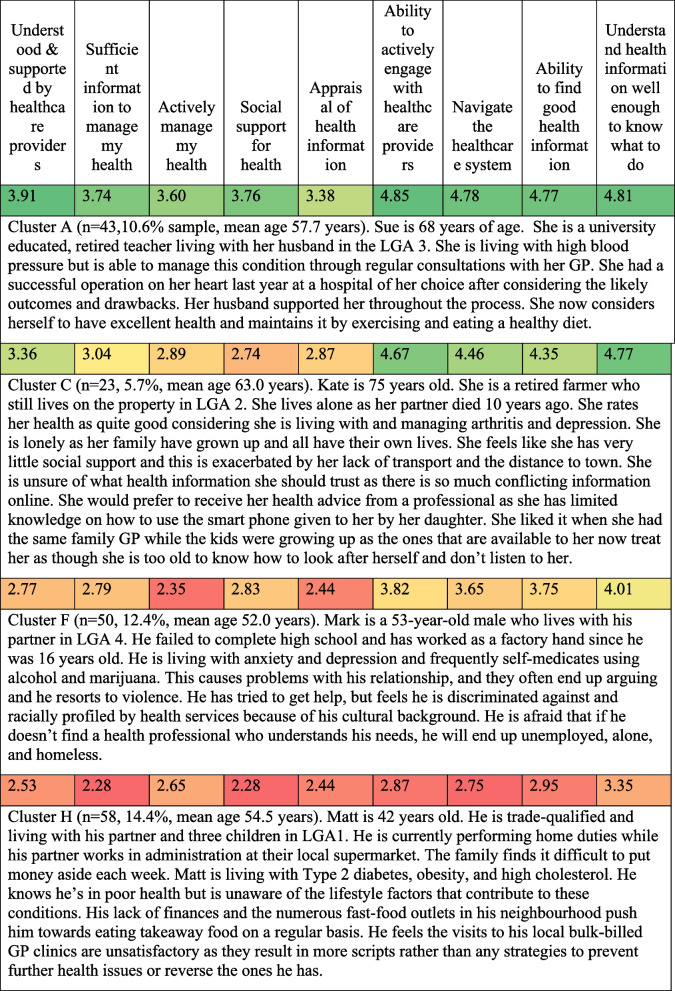
Health literacy profiles and vignettes for health literacy clusters for four local government areas

## Discussion

This year-long mixed-methods study was designed to achieve three ambitious aims, to:

1. Examine the intersection between health and local government to identify ways to better connect people to place-based PHC.
2. Better understand touch points for health in local government, particularly those related to Local Government Frameworks and regulation, for example the Public Health Act (2016).
3. Identify opportunities for localised reciprocal referral pathways between community organisations and PHC services.

The research identified issues that influenced PHC use in four local government areas in Western Australia, with common variables and themes identified during GMB processes resulting in combined CLD and prioritised action ideas. Systems science methodologies and community health literacy surveys were used to understand how to better connect people to place-based PHC and identify touchpoints for health from the perspective of community members and local organisations. In some circumstances community-led working groups were formed after the GMB to further develop and implement the action ideas. During the final stages of the project, the findings were presented to stakeholders who then identified opportunities for reciprocal referral pathways and better ways of working together. Although place specific, many of the findings are similar as so are likely to be transferable across other metropolitan LGAs in Western Australia.

A recurrent theme across all LGAs was the urgent need for greater access to services, the findings suggest that this could be achieved by building the health literacy of community members and service providers, and by creating more inclusive services. At the GMB workshops some participants were surprised to learn of the types of services that were on offer locally, suggesting a need to promote them. This information is likely to increase access to services and may assist health care practitioners in developing treatment plans and local referral pathways. Greater access would be facilitated by outlining culturally specific capabilities of services, for example, the languages spoken by practitioners, whether Cultural Safety Education and Action training had been undertaken, availability of female practitioners, and areas of speciality (e.g. mental health, sex specific health care, child health, etc.). Overcoming barriers such as poor or no internet connection, low levels of digital literacy, or lack of technology could also increase health service access, LGAs and local service providers could work together to offer internet access, education in digital literacy, and alternate ways to book services, and local librarians could upskill older people, those with disabilities, and help with the technology or accessing data.

Findings suggest that the provision of outreach services and facilitating transport may assist with service access for some community members. Outreach or pop-up services reduce transport needs as does co-location of services within the local community centres. People said they would feel more comfortable seeing mental health counsellors while attending health at local shopping centres or other community locations. Regularly offering medical checks at local seniors’ clubs, for example blood pressure, or blood glucose, were reported as successful in one LGA. Youth hangouts in underutilised local government spaces was another proposition embraced by community members. Our survey showed that these types of local touchpoints were supported by 83% of respondents who indicated that they would be likely to attend a health screening at a local government facility such as a library.

Vulnerable groups, people accessing mental health or disability services, those from culturally and linguistically diverse backgrounds, and older people were a priority consideration in each LGA, and there was a strong perception that these groups were misunderstood or dismissed by health care practitioners, leading to further service exclusion and referrals to inappropriate treatment pathways. The lack of knowledge of health services, allied health and other community services was a major concern across all LGAs.

The findings support the notion that local community organisations can play a role in reducing or preventing some of the common determinants of mental health and chronic disease through methods such as social prescribing [37]. This approach provides healthcare that is person-centred, holistic and acknowledges the social determinants of health. Social prescribing has been shown to reduce emergency department usage, inpatient admissions, general practice over-attendance and reduce GP workload in some cases but the evidence is mixed [38–40]. This type of strategy may go some way to alleviating the waiting lists and lack of services, including the shortage of GPs outlined during the consultations, however, further research is necessary. This type of non-medical prescription to improve health and wellbeing can include referrals to social activities, sources of information or guidance, and skills development opportunities. The facilitation of partnerships and collaboration at the local level is a key strategy to address health and wellbeing. There was evidence during the GMB discussions of this type of opportunity being used to build mental wellbeing, for example, through partnership with the Act Belong Commit mental health promotion campaign which increases awareness of the importance of the link between social connection and mental health [41]. The campaign subscribes to Margaret Barry’s (2019) view that “mental health promotion cannot be undertaken by any one sector or any single organization on its own” pg 81[42] and World Health Organization’s recommendation that that different sectors and organisations work together to impact public health [43]. The Act Belong Commit campaign’s message to Act (do something), Belong (do something with someone), and commit (do something meaningful) [44] has been implemented in a number of countries [45, 46], as it provides a mechanism for numerous organisations to work together to promote mental wellbeing [47]. Discussions across all LGAs revealed that organisations such as Men’s Shed [48], the local service clubs, clubs for seniors, multicultural groups, and faith-based groups provide social support and could be used by local primary care practitioners in community referrals. Participation in these social groups can help break the cycle of isolation, loneliness and depression. However, just referring people to these groups and activities may not be enough as lack of appropriate transport was a major barrier across LGAs. Community buses, or volunteers driving their private cars were raised as possible solutions.

Local sports clubs and exercise groups help reduce the burden of chronic disease as they provide opportunities to exercise and to socialise. The local seniors exercise group in one LGA has been running for many years helping members to build new friendships, gain social support and grow a strong sense of community. There was concern about the sustainability of this, and groups like it, as they rely heavily on ageing volunteers, with few younger people ready to take their place. Despite the benefits to the individual of volunteering[49], the declining number of volunteers was commonly cited by organisations, that has increased since the COVID-19 pandemic [50]. Additionally, consistent with existing evidence, volunteers in these LGAs report fatigue leading to stress, burnout and associated problems [51].

Mental health was identified as a key issue by all LGAs, as was the need to build the appropriateness and availability of mental health services at the local level. Shortcomings of mental health services were across the health care system, from insufficient access to locally based accessible counsellors and psychologists (with recommendations to increase the number of local specialists), a lack of skills among general practitioners (with suggestions to provide professional development),and prioritise mental health in tertiary education.

Preventive services were considered important, with specific recommendations to build local organisational capacity, for example, through establishing youth ‘hang out’ centres, local community activities to build wellbeing and foster and maintain connections and co-locating services within existing local government facilities. There was a shared understanding that these actions were presented as ways to halt and reduce the ever-increasing waitlists for counselling services.

The current study findings suggest that mental health literacy across the community needed to be improved, with one GMB group suggesting that mental health first aid training be made available to all community members and organisations. Given this training addresses mental health first aid, increases knowledge regarding recognition and treatment of mental disorders, and addresses stigma and improving confidence in engaging with people with mental health issues [52], this training would likely be benefit all communities in this study. However, given the complexity of issue relating to mental health identified in this study (including domestic violence, economic hardship, alcohol and other drugs, discrimination, and stigma), targeted training is recommended. Also, there have been recent changes in workplace legislation around domestic violence and bullying [53] and this information should be incorporated into mental health first aid training.

For many reasons, community members felt there was a need to build trust in the health system. The racism, stigma, culturally inappropriate, and discriminatory practices were described as negative experiences leading to vulnerable people being less likely to be engaged with health services. Racism has long been recognised as a social determinant of health in Australia with evidence that culturally respectful health care delivery can lead to improvements in the short term, but that more is needed to reduce disparities in the long term [54, 55]. One group recommended that the GMB process be conducted with a group of Aboriginal and Torres Strait Islander people to address this issue, this is consistent with recommendations to build HL through a two-way capacity (Aboriginal people and health service providers) [55].

Community health literacy scores were of a level similar to those published in recent Australian studies [36, 56]. The lowest health literacy scores were in the scales related to the ability to actively manage one’s own health, to appraise health information, having social support for health, having sufficient information to manage health, and feeling understood and supported by healthcare practitioners. These findings were also confirmed in the CLDs reported in this study. Socio-demographics play an important role in determining levels of health literacy. Cluster profiles demonstrated the associations between health literacy and education, paid employment, and ability to save money, and this is consistent with a recent literature review which found that there were associations between social gradients and health literacy in all analyses of national population surveys [57]. Low levels of health literacy have also been found to be associated with being a male from a racial/ethnic minority, who is highly influenced by family or significant other, and is unable to work [58]. Among a sample of Australian First Nations people, high health literacy levels were related to being less than 55 years old, female, having only one chronic disease, higher levels of education and an income of less than AUD$55,000 [59].

The current study findings suggest there is a need to improve health literacy among vulnerable target groups and to understand local issues. Feedback sessions with health service and local government organisations suggested that the vignettes would be particularly useful for them in helping them to describe the types of people who should be targeted in any intervention, and the areas that already have high levels of health literacy. The locally identified and prioritised action ideas identified by those attending the workshops will require resourcing and support to reach fruition. Follow-up workshops with key stakeholders identified that some of the services recommended already existed, and this confirmed the need for ongoing collaboration and development of communication mechanisms to sharing information at the local level. The final outcomes of the research were reported to funders with key recommendations, based on the findings, emphasising the expectations of participants to changes in service delivery as a result of the consultation.

Finally, this research applied a systems mapping approach as the purpose was to try to understand this complex, adaptive system in a practical, actionable, and participatory way. The mapping resulted in a real-world, shared understanding of causal relationships described at a local level in four different communities. This paper presents the common themes, there were also some local differences. The time taken to build relationships and engage local stakeholders was needed, and it was valuable placing researchers in each organisation to assist with this engagement. The research aimed to consider the intersection between health, health literacy and local government to identify ways to better connect people to place-based primary health care (PHC).

Barnrook-Johnson and Penn (2022) assert in there reflections on systems mapping, that “There is a growing need in a range of social, environmental, and policy challenges for a richer more nuanced, yet actionable and participatory, understanding of the world” pg vii [60], and this current study using a systems approach coupled with the measurement of health literacy provided rich insights and delivered community driven place-based priorities actions.

### Strengths and limitations

A strength of this research is that we had wide consultation with community members, we used several data sources across four LGAs. Researchers were embedded within each of the areas for about a year which built strong relationships and facilitated engagement with both the LGAs, community members and organisations. A limitation of this research was that it was a scoping exercise, rather than participatory research to develop and implement interventions. Several of the community action ideas identified could be developed further and implemented at the local level however, would require collective impact which needs organisational support and funding [61]. Although explicitly stated at the initial workshop that this was not the case, the consultations and engagement created expectations of future support and action. This may have impacted on the willingness of people to engage in the ongoing process. It is important not to over consult, rather, projects like this one should focus on collaboration and dissemination of findings across relevant sectors.

## Conclusion

There are many possibilities for health care and local governments to work together to bring services to community members disengaged from the health system. Bringing people from diverse backgrounds and organisations together created synergies that resulted in novel and feasible potential strategies to improve to improve community health. Social prescribing is one strategy that could be activated to reduce the strain on an already overburdened health system.

## Data Availability

The datasets used and/or analysed during the current study are available from the corresponding author on reasonable request.

## Abbreviations

CLD: Causal Loop Diagram
GMB: Group Model Building
HL: Health Literacy
LGA: Local government areas
WA: Western Australia
PHC: Primary health care
